# Alveolar soft part sarcoma metastatic to the breast: a case report

**DOI:** 10.1186/s12893-019-0494-8

**Published:** 2019-03-04

**Authors:** Yuka Asano, Shinichiro Kashiwagi, Koji Takada, Sadao Tokimasa, Tsutomu Takashima, Masahiko Ohsawa, Kosei Hirakawa, Masaichi Ohira

**Affiliations:** 1 Department of Breast and Endocrine Surgery, Osaka City University Graduate School of Medicine, 1-4-3 Asahi-machi, Abeno-ku, Osaka, 545-8585 Japan; 2 Department of Pediatrics, Osaka City University Graduate School of Medicine, 1-4-3 Asahi-machi, Abeno-ku, Osaka, 545-8585 Japan; 3 Department of Diagnostic Pathology, Osaka City University Graduate School of Medicine, 1-4-3 Asahi-machi, Abeno-ku, Osaka, 545-8585 Japan

**Keywords:** alveolar soft part sarcoma, mammary tumor, surgery, FDG-PET, metastatic breast tumor

## Abstract

**Background:**

Alveolar soft part sarcoma (ASPS) is an extremely rare neoplasm that tends to occur in the lower limbs of children and adolescents. Metastatic breast tumors constitute 0.5–2.0% of all malignant mammary neoplasms, and cases of ASPS with mammary metastases are very rare.

**Case presentation:**

Three years ago, an 11-year-old girl presented to the hospital with pain in the right jaw after becoming aware of a mass in the right cheek. After detailed examination, the patient was diagnosed with ASPS with the primary tumor in the right cheek and multiple lung metastases, and chemotherapeutic treatment was initiated. One year later, accumulation of fluorodeoxyglucose (FDG) was observed in the right front of the skull (standardized uptake value (SUV)-max 2.8) and left breast (SUV-max 2.4) using FDG-positron emission tomography (PET) / computed tomography (CT). Ultrasonography revealed the mammary tumor as a hypoechoic, internally heterogeneous mass measuring 22.4 × 16.2 × 21.1 mm with a rich blood supply. Using pathological findings of core-needle biopsy, we diagnosed it as ASPS. Based on the above information, we made a diagnosis of ASPS with left mammary and cranial metastases. Due to chemoresistance, surgical excision was selected as the mode of treatment; resection of the metastatic cranial bone was performed first, and partial mastectomy of the left breast was performed in two stages. Postoperative conditions were good, and we are currently performing regular follow-ups (visual palpation every 3 months and semi-annual mammary gland ultrasonography).

**Conclusions:**

We have reported an extremely rare case of ASPS with mammary metastasis with some reference-based discussion. In our case, disease control was obtained by a combination of drug therapy and surgical treatment.

## Background

Alveolar soft part sarcoma (ASPS) is an extremely rare neoplasm that tends to occur in the lower limbs of children and adolescents and was first described by Christopherson et al. in 1952 [1, 2]. Metastatic breast tumors constitute 0.5–2.0% of all malignant mammary neoplasms [3, 4], and cases of ASPS with mammary metastases are very rare [5–7]. Here, we report a case of ASPS with mammary metastasis with some reference-based discussion.

## Case presentation

**Fig. 1:**
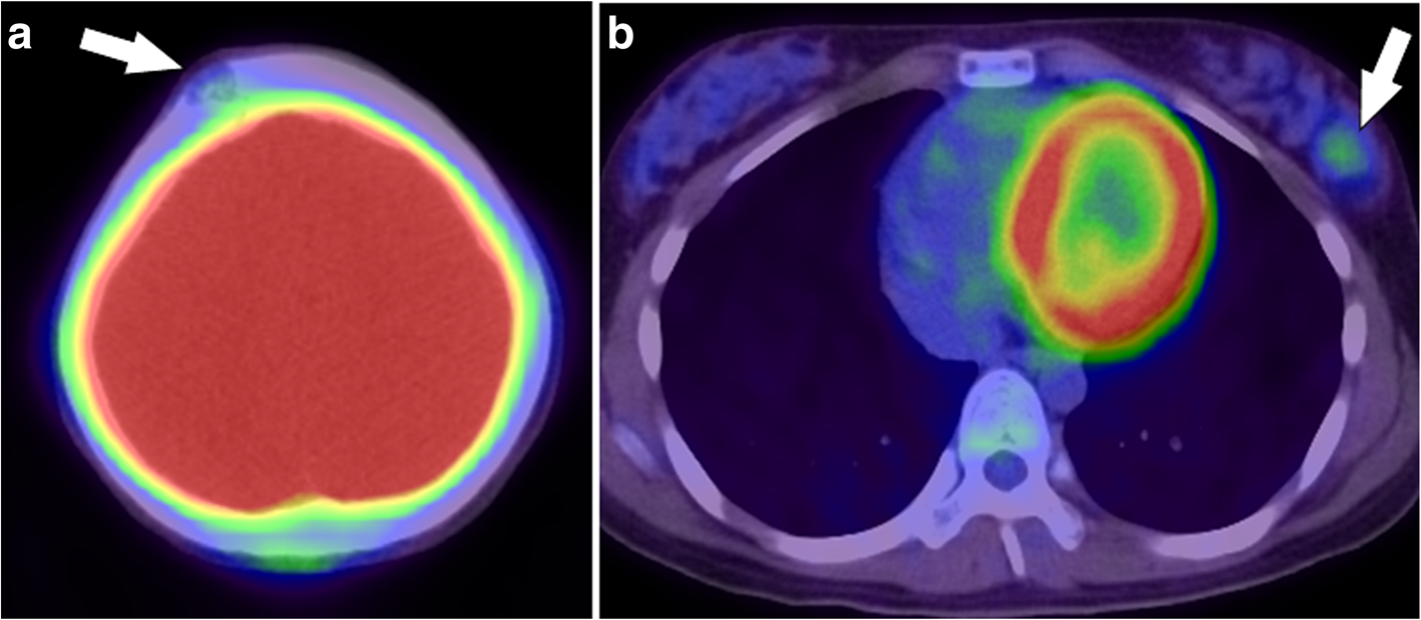
FDG-PET / CT image findings: Accumulation of fluorodeoxyglucose (FDG) was observed in the right front of the skull (standardized uptake value (SUV)-max 2.8) (**a**) and left breast (SUV-max 2.4) (**b**) using FDG-positron emission tomography (PET) / computed tomography (CT)

Three years ago, an 11-year-old girl presented to the hospital with pain in the right jaw after becoming aware of a mass in her right cheek. After detailed examination, the patient was diagnosed with ASPS with primary tumor in the right cheek and multiple lung metastases, and chemotherapeutic treatment was initiated. After receiving 1 cycle of VAC therapy (vincristine [2 mg], actinomycin D [0.045 mg/kg], and cyclophosphamide [1.2 g/m^2^]), the patient developed grade 4 neutropenia. After this treatment, the patient received 1 cycle of the treatment regimen prescribed for rhabdomyosarcoma (vincristine [2 mg], pirarubicin [60 mg/m^2^], cyclophosphamide [1.2 g/m^2^], cisplatin [20 mg/m^2^]) and 1 cycle of ifosfamide (1800 mg/m^2^), etoposide (100 mg/m^2^), actinomycin D (0.045 mg/kg), and vincristine (2 mg); however, the development of severe neutropenia made it difficult to continue administration of these drugs. The patient was then treated with oral administration of 800 mg/day of pazopanib for 1 year, and clinical benefit was achieved. Upon stabilization of the disease, oral administration of pazopanib was discontinued; however, 1 year later, fluorodeoxyglucose accumulation was observed in the right front of the skull (maximum standardized uptake value [SUV-max], 2.8) (Fig. 1a) and in the left breast (SUV-max, 2.4) (Fig. 1b) using fluorodeoxyglucose-positron emission tomography/computed tomography.

**Fig. 2:**
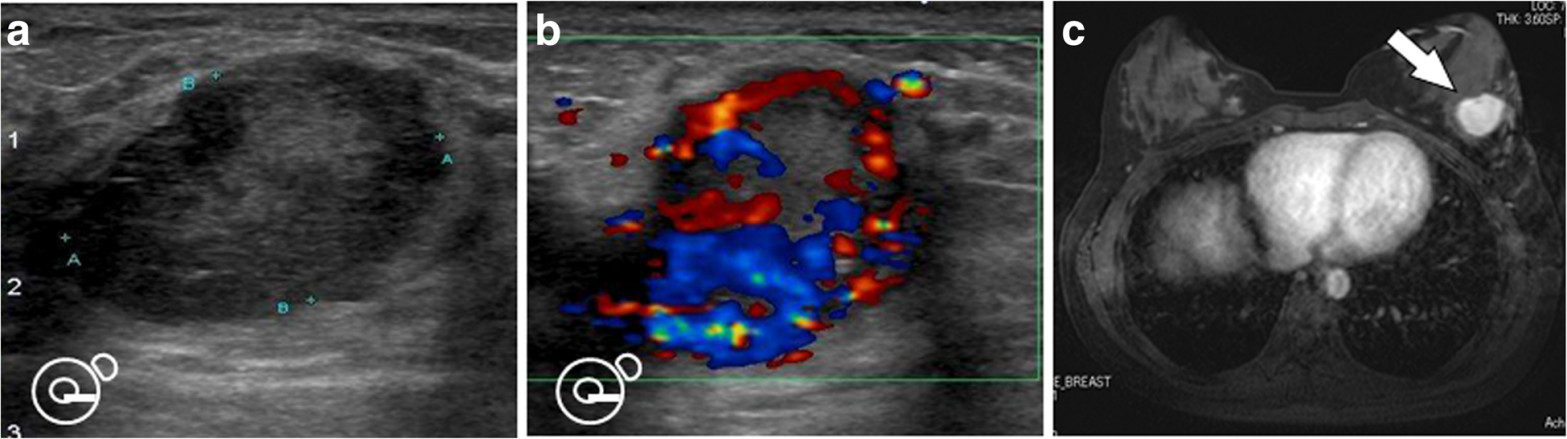
Ultrasonography and MRI image findings: The mammary tumor was revealed as a hypoechoic, internally heterogeneous mass measuring 22.4 × 16.2 × 21.1 mm (**a**) with a rich blood supply (**b**) using ultrasonography, while magnetic resonance imaging findings identified a 3-cm tumor larger than that found on prior imaging (**c**) (arrow)

**Fig. 3:**
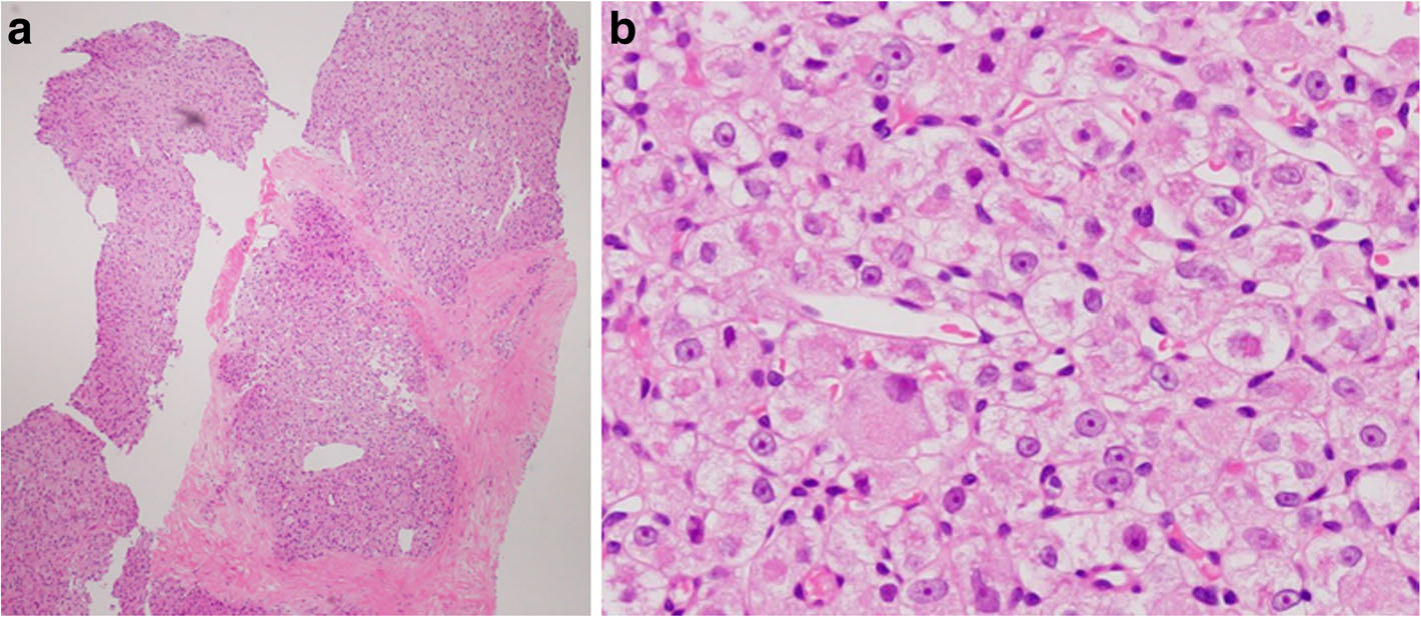
Pathological diagnosis of core-needle biopsy specimen (Hematoxylin and eosin stain): Tumor cells proliferating to form a solid tumor with comparatively abundant foamy cytoplasm, clear nucleoli, and oval nuclei were found in a core-needle biopsy specimen (**a**: × 40) (**b**: × 400)

**Fig. 4:**
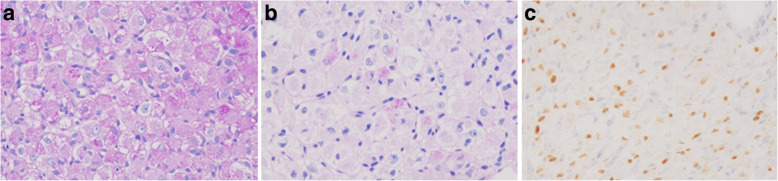
Pathological diagnosis of core-needle biopsy specimen (Immunohistochemistry): The specimen negatively converted after diastase digestion of periodic acid–Schiff (PAS) staining (**a**, **b**), which was TFE3-positive (**c**) (× 400)

**Fig. 5:**
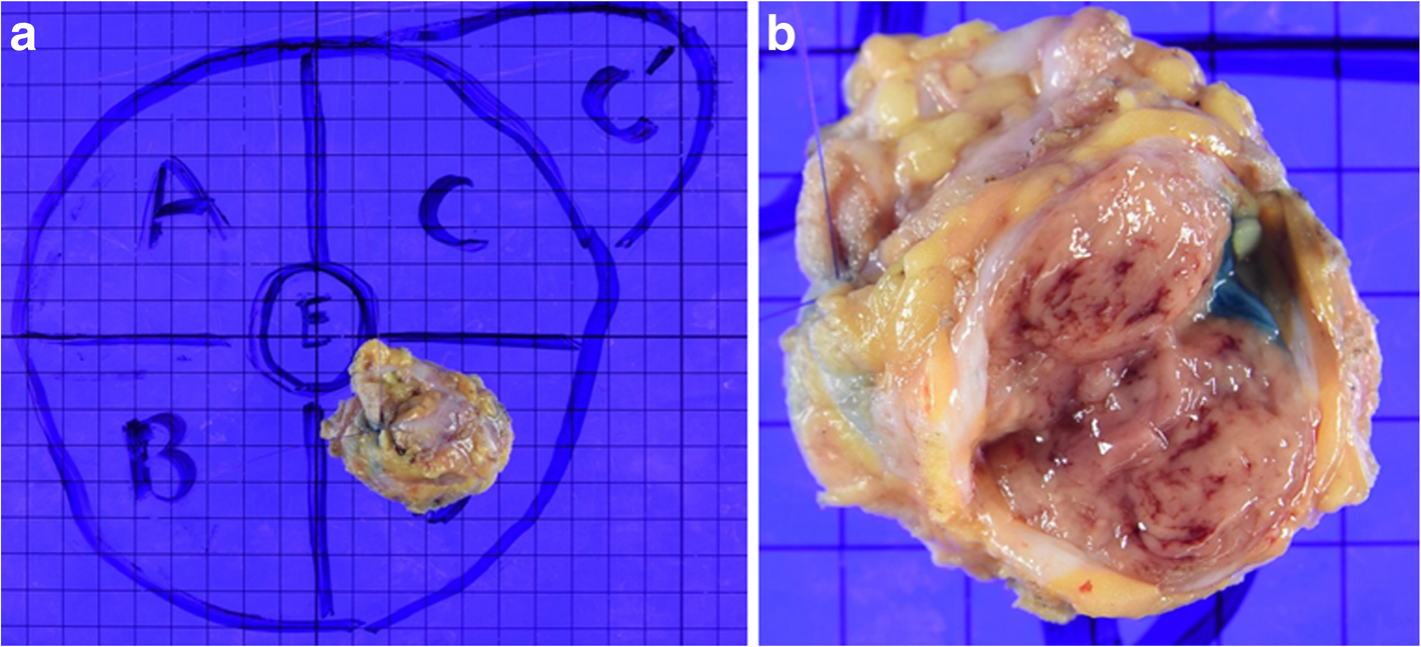
Macroscopic diagnosis of the resected specimen: The mammary tumor was 25 mm in size (**a**), and the cut surface was solid with a reddish gray color (**b**)

An elastic, soft tumor, approximately 3 cm in size, was palpated in the lower lateral region of the left breast. Ultrasonography revealed a hypoechoic, internally heterogeneous mass measuring 22.4 × 16.2 × 21.1 mm with a rich blood supply (Fig. 2a, b), while magnetic resonance imaging showed a 3-cm sized tumor that was larger than the one found on prior imaging (Fig. 2c). Examination of a core-needle biopsy specimen from the same site showed proliferating tumor cells with abundant foamy cytoplasm, clear nucleoli, and oval nuclei (Fig. 3a, b). The tumor cells tested positive for AE1/AE3, CAM 5.2, vimentin, S-100, α-actin, desmin, and HMB 45. The specimen showed negative periodic acid–Schiff (PAS) staining after diastase digestion (Fig. 4a, b); furthermore, the specimen then tested positive for transcription factor E3, resulting in a pathological diagnosis of ASPS (Fig. 4c). Based on the above information, we established a diagnosis of ASPS with left mammary, lung, and cranial metastases. Due to chemoresistance, surgical excision was selected as the mode of treatment; resection of the cranial bone showing metastasis was performed first and partial mastectomy of the left breast was performed in two stages. The mammary tumor was 25 mm in size, and the cut surface was solid with a reddish gray color (Fig. 5a, b). Histological findings similar to those of the needle biopsy specimen were also obtained in the final pathological diagnosis and resection margins were negative. Postoperative conditions were good, and we are currently monitoring the patient through regular follow-ups (visual palpation every 3 months and semi-annual mammary gland ultrasonography).

## Discussion and conclusions

Metastatic tumors of the breast are rare and account for approximately 0.5–2.0% of all malignant mammary tumors [1]; however, they are successively more prevalent in malignant lymphomas, malignant melanomas, and lung cancer [4]. It has been reported that the rarity of metastases to the mammary glands may be due to the recession and replacement of mammary gland tissues with connective or adipose tissues at the age when cancer development is most likely; the corresponding reduction in blood flow may also be responsible. Clinical findings in such cases often involve a singular mass commonly occurring in the upper lateral part of the breast with no skin involvement or pain, and no overt differences from primary breast cancer are observed. Imaging findings are similar to those of benign tumors [8]; however, the lack of ductal or lobular tumors in pathological diagnosis and retention of the intrinsic structure of the mammary gland are the key differences between benign and metastatic tumors of the breast [9].

Conversely, ASPS is rare and accounts for 0.5–1.0% of soft-tissue sarcomas; it commonly occurs in young women. In adults, it is often found in the lower limbs, while it commonly occurs in the head and neck in children [2, 10]. At the time of definitive diagnosis, 60–70% of cases involve distant metastases to the lungs, bones, and brain, and the condition has a poor prognosis with an average survival time of approximately 40 months. Cases with ASPS metastasis to mammary tissue are considered extremely rare and are reported only in 10 previous studies [5, 6, 11]. Orphans et al. [7] reported no difference between the left and right onset (11~29-year-old) in the case of breast metastases from ASPS, and about half of the cases were multiple organ metastases (number of lesions: range 1~3, median 1.5). In our case, we observed juvenile onset and multiple organ metastases. Since the size of the tumor were relatively small, about 2 cm, we performed partial resection of the mammary gland. Pathological histology in such cases is characterized by an alveolar proliferation of atypical cells with circular nuclei and clear cell bodies, intracellular periodic acid–Schiff positivity of the atypical cells, and presence of diastase-resistant and non-resistant glycogen granules [12, 13]. Similar pathological findings were also observed in our case, except that diastase-resistant granules were absent. However, positive expression of transcription factor E3 was confirmed, which is considered to be a pathological characteristic of ASPS. In ASPS, assessment of t (X; 18) translocation using fluorescence in situ hybridization or karyotyping is regarded as the standard diagnosis; however, this test was not used in our case.

Surgical excision is most commonly used for treatment because of chemotherapeutic resistance [14, 15]. In recent years, a number of reports have indicated the efficacy of the molecular targeted drugs, pazopanib and sunitinib [16, 17], and future development is expected. Pazopanib might not cross the blood-brain barrier and this may be the reason for tumor regrowth in the brain despite treatment with this drug.

We reported an extremely rare case of ASPS with mammary metastasis with some reference-based discussion. In our case, disease control was obtained by a combination of drug therapy and surgical treatment.

## Abbreviations

ASPS: Alveolar soft part sarcoma
CT: Computed tomography
FDG: Fluorodeoxyglucose
PAS: Periodic acid–Schiff
PET: Positron emission tomography
SUV: Standardized uptake value
TFE3: Transcription factor E3
